# Bayesian aggregation versus majority vote in the characterization of non-specific arm pain based on quantitative needle electromyography

**DOI:** 10.1186/1743-0003-7-8

**Published:** 2010-02-15

**Authors:** Andrew Hamilton-Wright, Linda McLean, Daniel W Stashuk, Kristina M Calder

**Affiliations:** 1 School of Rehabilitation Therapy, Queen's University, Kingston, Ontario, Canada; 2 Math and Computer Science, Mount Allison University, New Brunswick, Canada; 3 Computing and Information Science, University of Guelph, Ontario, Canada; 4 Department of Systems Design Engineering, University of Waterloo, Ontario, Canada

## Abstract

**Background:**

Methods for the calculation and application of quantitative electromyographic (EMG) statistics for the characterization of EMG data detected from forearm muscles of individuals with and without pain associated with repetitive strain injury are presented.

**Methods:**

A classification procedure using a multi-stage application of Bayesian inference is presented that characterizes a set of motor unit potentials acquired using needle electromyography. The utility of this technique in characterizing EMG data obtained from both normal individuals and those presenting with symptoms of "non-specific arm pain" is explored and validated. The efficacy of the Bayesian technique is compared with simple voting methods.

**Results:**

The aggregate Bayesian classifier presented is found to perform with accuracy equivalent to that of majority voting on the test data, with an overall accuracy greater than 0.85. Theoretical foundations of the technique are discussed, and are related to the observations found.

**Conclusions:**

Aggregation of motor unit potential conditional probability distributions estimated using quantitative electromyographic analysis, may be successfully used to perform electrodiagnostic characterization of "non-specific arm pain." It is expected that these techniques will also be able to be applied to other types of electrodiagnostic data.

## Background

It is generally accepted that non-specific arm pain (NSAP) is caused by physical exposures in the workplace including repetitiveness, awkward postures, and high forces, and this condition is commonly reported in the workplace [1]. In a 2-year prospective population based cohort study with retrospective assessment of exposures at work, Macfarlane *et al *[2] found mechanical factors moderately increased the risk of NSAP, with repetitive motion being the most important factor for the onset of pain. However, a study by Walker-Bone *et al *[3] found that individuals with NSAP were no more likely to develop a known pathology, such as hand-wrist tendonitis from repetitive keyboard work, than individuals without underlying forearm pain, suggesting that the diffuse pain felt in NSAP is not simply a precursor to a more clearly defined musculoskeletal condition.

Despite known risk factors, little is known about the pathology of NSAP, where the diffuse pain noted in the forearm of affected individuals lacks any clear diagnostic criteria. In fact, the Harrington criteria [4] define non-specific forearm pain as a pain in the forearm that fails to meet the diagnostic criteria for other specific diagnoses and/or diseases.

It is not clear whether NSAP is a musculoskeletal or neuromuscular condition. Some authors believe that chronic pain conditions like NSAP and trapezius myalgia are associated with damage within the muscle [5-10], whereas others believe it is caused by neuropathic changes [11-14]. In some muscles affected by chronic overuse conditions, an increased proportion of "ragged red" fibers have been identified on biopsy as compared to healthy control subjects, and researchers have therefore suggested that the origin of this condition is associated with mitochondrial damage to the Type I fibers [15-17], however these results have not been conclusive, with similar damage noted in individuals who perform repetitive tasks but who are pain free. Other researchers have found indications that chronic muscle pain in the wrist flexor group (also referred to as NSAP) may be neuropathic in nature [11-14]. In particular, Greening *et al *speculate that NSAP affecting the wrist flexor muscles is neuropathic in origin, based on observed changes in median nerve function [11,12,18].

### Quantitative electromyography

Quantitative electromyographic (EMG) data can be used to obtain reproducible and robust characterizations of the signature signal structures obtained from individual motor units (MUs) [19,20]. Through signal decomposition techniques applied to a needle-detected EMG signal, it is possible to observe the repeated occurrence of motor-unit potentials (MUPs) from the pool of motor units active during a given muscle contraction. The series of such potentials is referred to as a motor-unit potential train, or MUPT; these data may be used to characterize both the average shape of a MUP as well as to estimate the firing pattern of its generating MU. In addition, by combining data simultaneously acquired using surface and needle electrodes, it is possible to correlate the data from these sources and obtain an estimate of the surface representation of the MUP (called an SMUP template) related to each MUPT. The SMUP is determined by using the firing times of the main spike of each individual MUP firing within a MUPT and relating these to the potential observed at a surface electrode overlying the needle uptake volume. By considering a "window" based on the needle-triggered firing, a template of the mean observed voltage may be constructed by ensemble averaging the voltages for each sample across the window associated with each firing. This will produce a template, seen at the surface electrode, of the average voltage shape related to the needle-observed MUP.

Through aggregate analysis of the MUPTs detected during a contraction, or set of contractions, it is possible to obtain information about the active MUs within a muscle. This work provides an analysis of the information obtained through an aggregation approach.

The MUPTs considered were detected in the forearm muscles of individuals with and without NSAP. By using a simple, statistically based, Bayesian classification algorithm, we wished to explore the degree to which estimates of the multidimensional distributions of features used to represent MUPTs may be used to classify sets of MUPTs, and to differentiate subjects with NSAP from pain free subjects.

Each MUPT may be considered to have a characterization. In this work, a MUPT characterization is defined as a set of two conditional probabilities: that of being detected in a muscle of a subject with NSAP and that of being detected in a muscle of a subject free of pain. If we maintain our understanding of this MUPT characterization in purely probabilistic terms, then by considering a set of MUPTs detected from the same muscle we may estimate the overall conditional probability that the muscle is from a subject with NSAP versus the probability that the subject does not. This overall conditional probability will be based on more evidence than is available by analysis of an individual MUPT. Each MUPT contributes its conditional probability as a weighted vote toward each possible class labelling.

Bayesian aggregation has been used in several fields [21-25], including various medical and clinical applications [26,27]. Pfeiffer [28,29] first proposed Bayesian aggregation as a technique for combining the clinical information available from the analysis of multiple motor unit potentials. Bayesian aggregation considers *a priori *information about data distribution shapes and relative numbers of occurrence and combines it with specific sampled data values to produce an overall characterization. Our intention here is to explore this technique in relation to the poorly understood problem of NSAP, and evaluate the utility of the Bayesian technique.

NSAP is of interest in a diagnostic sense as the underlying pathophysiology is unknown; we therefore propose a test that is discriminative for this condition. Based on quantitative EMG data analysis, it is hoped that some insight into the morphological differences seen in MUPTs detected in muscles of subjects with NSAP, and thus its pathophysiology, may be obtained.

It should be noted, however, that as in any similar condition, a large enough sample of MUPTs from an affected individual would contain MUPTs consistent with the involved state, as well as essentially normative MUPTs. This is due simply to the fact that it is unlikely that the condition has a uniform effect on all motor units sampled; while some units will potentially be quite significantly involved, other units may be free of any involvement at all. The MUPTs associated with these uninvolved units will therefore produce measures that are consistent with normative values, and their presence in data acquired from an involved subject will make correct interpretation more difficult. It is therefore reasonable to hypothesize that both normative and involved MUPTs will be acquired from the same muscle (indeed, during the same contraction), and that there is no clear way to definitively separate such MUPTs using any type of gold-standard as both may be considered to be representative of a specific condition.

This situation is not restricted to NSAP. One must, in fact, assume that this problematic condition may be present in any type of diagnostic data related to a process with variable involvement. As involvement proceeds, it may be expected that more and more of the data obtained in a sample may indicate a specific condition, however it is unlikely that all samples may be considered unequivocally indicative of the condition, except in very extreme cases.

## Methods

### Data collection

Ethics approval for this study was obtained from the Queen's University Health Sciences Research Ethics Board. Electromyographic (EMG) data were collected from 17 volunteers with signs and symptoms consistent with NSAP, as well as a normative group of 40 volunteers.

A clinical examination was performed and used to make demographic comparisons between the groups, to verify correct group assignment, and to verify that subjects had no signs or symptoms of cervical radiculopathy and/or other repetitive strain injury such as carpal tunnel syndrome, deQuervain's tendonitis, or medial epicondylitis. The screening examination consisted of a neurologic examination of the upper extremities, including myotome testing, dermatome (light touch, pin prick) testing, and assessment of the deep tendon reflexes at the C5 to C8 levels. Cervical spine range of motion was tested in sitting to ensure that cervical movements did not reproduce the forearm symptoms. The movements tested included flexion, extension, lateral flexion, rotation, and combined extension with lateral flexion. These movements were held at the end of the available range of motion for 10 seconds. Three repetitions of maximal handgrip strength (Jamar Dynamomter, Sammons Preston Inc., Model # 5030J1; in position 2) and maximal pinch grip strength (Baseline Evaluation Instruments, 60# mechanical pinch gauge, model # 12-0201) were measured bilaterally with the elbow flexed to 90 degrees, and with the wrist held in neutral between flexion and extension, respectively.

For the participants in the NSAP group, several other parameters were recorded and were used as a basis for comparison for other samples not presented here. See [30] for details.

A pressure algometer (model PTH-AF 2, Pain Diagnostic and Treatment Corporation, Great Neck, NY 11021, USA) was used to measure pain pressure threshold (PPTh) and pain tolerance (PPtol). The device consists of an analog force gauge fitted with a disc-shaped rubber tip (1 cm^2^). The range of the gauge is 0-10 kg, with increment markings at 0.1 kg. Measurements were made at the nail bed of the third digit (D3), over the bellies of the extensor carpi radialis brevis (ECRB) muscle, the flexor carpi radialis (FCR) muscle, the biceps brachii (BB) muscle and the triceps brachii (TB) muscle. Pain tolerance scores (PPtol) were normalized to the amount of pressure subjects could withstand having applied to the nail bed on D3 of the affected (or tested) limb.

Subjects who were assigned to the NSAP group experienced pain on palpation of the ECRB muscle and complained of forearm pain during wrist extension activities performed at work or in their leisure activities, but resisted wrist extension with elbow extension as described above did not reproduce their signs and symptoms. We did not include any subjects who had signs or symptoms that could be attributed to lateral epicondylitis (*i.e*.; pain on resisted extension of digit 2 or 3, or pain on passive wrist flexion with the elbow extended). Control subjects had no pain on resisted wrist extension, passive wrist flexion, or palpation of the lateral epicondyle or the ECRB muscle. Subjects in the control group did not perform repetitive wrist motions at work or during their leisure time. Both subject groups excluded individuals with known cardiovascular, metabolic (diabetes) or neurologic disorders. All subjects provided informed consent prior to participation.

**Figure 1:**
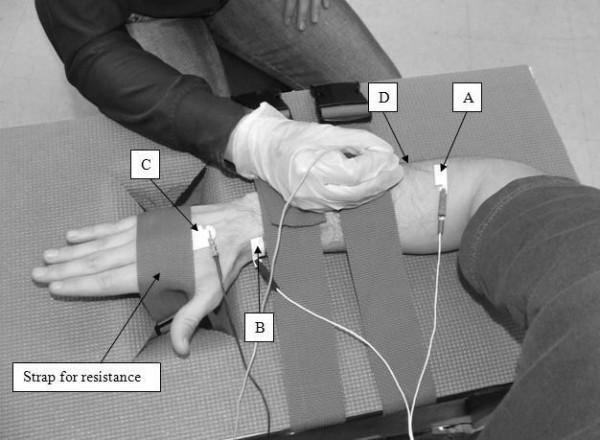
**Data Collection Procedure**.

For the electromyographic evaluation, subjects were seated in a straight back chair with the elbow of the dominant arm flexed at 90° and their forearm pronated and resting on a custom-built table (Figure 1). Adjustable straps attached to the bottom of the testing table were passed through an opening and secured around the dorsum of the hand to provide resistance during the isometric extension contractions. Surface electrodes (Ag/AgCl; Kendall-LTP, Chicopee, Massachusetts, cut in half to measure 1 × 3 cm) were placed on the tested limb, and subjects were asked to perform a three second maximum voluntary contraction (MVC) of their wrist extensors with verbal encouragement provided throughout. The peak root mean square (RMS) value calculated over contiguous one second intervals of the surface EMG attained during the MVC was determined. This value represented the maximal voluntary EMG produced by the subjects, termed maximal voluntary effort, or MVE. The RMS values of all subsequent contractions were expressed as a percentage of this value, and are referred to as the %MVE-RMS.

Quantitative EMG analysis was performed using the DQEMG method and associated algorithms. These were used as described in detail elsewhere [30-32]. Prior to electrode placement, the motor point of the ECRB muscle of the test limb was identified as the area over the muscle surface where the lowest possible electrical stimulus produced a muscle twitch. The location of the motor point in the ECRB muscle is approximately two cm distal to the cubital crease. Using the cathode portion of a stimulating probe, with the train rate of the stimulator set at 10 pps, and the stimulation duration set at 1 ms [33], the cathode was moved over the muscle belly until the motor point region was determined. The skin above the motor point, the radial styloid process and the dorsum of the hand of the test limb was cleaned with rubbing alcohol prior to electrode placement. The active electrode was positioned over the motor point of the ECRB and the reference electrode was placed over the radial styloid process to form a monopolar configuration, as described in [19]. A full-sized surface electrode (2 cm by 3 cm) was positioned on the dorsum of the hand to act as the common reference. A disposable concentric needle (Model 740 38-45/N; Ambu^® ^Neuroline, Baltorpbakken, Ballerup, Denmark) electrode was inserted approximately 2 cm deep underneath the active surface electrode.

AcquireEMG algorithms running on a Neuroscan Comperio EMG system (Neurosoft, Sterling, VA) were used to acquire the needle and surface EMG data during 30 s intervals as in [34]. The needle position was adjusted until the average peak acceleration of the MUPTs detected during a low-level contraction (5-10% MVE) was above 30 kV/s^2 ^[33]. Once a suitable needle position was found, the operator stabilized the needle manually and then asked the subject to hold a desired contraction force for 30 s. Subjects were provided with a visual bar graph and a numerical value that corresponded to their force output (%MVE-RMS) for feedback. Following each contraction the needle was moved (medially, laterally, superficially and/or deeper) so that MUPTs from different portions of the muscle would be sampled in an effort to record from a large representative pool of motor units. Each subject performed repeated contractions until at least 30 MUP trains were obtained. The contraction force was varied between 5-20% of MVE. A 2-minute rest period was provided between contractions.

The acquisition settings used were as reported in [30]: micro (needle) data were bandpass filtered between 10 Hz-10 kHz and then sampled at 31250 samples/second; macro (surface) data were a bandpass filtered between 5 Hz-5 kHz and sampled at 3125 samples/second.

### EMG decomposition

Needle-detected EMG data from all contractions were decomposed using the DQEMG program of Stashuk [32-34], which calculates a set of quantitative EMG summary statistics for each MUPT acquired during each muscle contraction. These measures describe the MUP shape and MU firing behaviour of each MU sampled from the muscle [35], and such parameters have been shown to be relevant in determining the type (myopathic vs. neuropathic) of disease involvement [28,29].

The DQEMG program produces a number of measures; the features used are listed in Table 1. These measures are common quantitative EMG parameters, the definition and collection of which are described in [19,35-37].

**Table 1:** Features Studied and their Units.

Transform	Feature	Abbreviation	Units
log	Amplitude	Ampl	ln(μV)
	Duration	Duration	μs
	Phases	Phases	
	Turns	Turns	
		
log	Area/Amplitude Ratio	AAR	ln(ms)
log	Macro Amplitude	Mac Ampl	ln(μV)
log	Macro Negative Peak Area	Mac. -Pk Area	ln(μV·ms)
log	Macro Neg. Peak Amplitude	Mac -Pk Ampl	ln(μV)
		
	Macro Negative Peak Duration	Mac -Pk Dur	ms
	Inter-Discharge Interval Mean	IDI mean	ms
	IDI Standard Deviation	IDI std. dev.	
	IDI Covariance	IDI cov	
		
	Inter-Discharge rate	IDRate	pps
	Firing Rate	FR	pps
	Firing Rate Mean Consecutive Difference	FRMCD	pps

A "log" Transform indicates that after the measurement of the feature, data was transformed using the natural logarithm before being used for calculation.

For some features, as noted in Table 1, logarithmic mapping was done in an attempt to provide a data distribution more closely approximating a Gaussian distribution, as many of the feature values stem from a multiplicative relationship between several underlying processes, causing their combined distribution to resemble an exponential distribution. Peak-to-peak amplitude is, for instance, a function of both the size and number of the active muscle fibres as well as the distance between these fibres and the electrode surface. As these factors combine multiplicatively, the distribution of observed values from a collection of fibres is extremely skewed, more closely describing an exponential distribution than a Gaussian one; the log of these values was therefore used to mitigate skewness. As skewness has serious implications for the classifier discussed later, this is expected to improve classifier performance; this hypothesis was confirmed through a set of preliminary experiments performed while preparing the data.

In the case of these log-transformed features, all calculations shown here were done with the log-transformed values.

### Data distribution construction and cross-validation

In total, 266 MUPTs were collected from the 17 subjects with NSAP and 1168 MUPTs were collected from the 40 control subjects. Each subject's EMG data set is henceforth referred to as a muscle study. Each muscle study is represented by the collection of the MUPTs extracted from EMG data detected from the same muscle during contractions performed on the same occasion. As the objective during data collection was to have at least 30 separately identifiable MUPTs for each muscle study, the number of contractions per study varied from subject to subject.

As mentioned in the introduction, the data in the NSAP class contains several samples that would and should be considered normative, greatly increasing the difficulty of the characterization task. One of the major outcomes of this analysis is to show to what degree it is possible to aggregate the information from MUPTs with a variety of individual characterizations, across a set of MUPTs, to produce a correct overall characterization of a muscle as being either NSAP or normative.

In order to establish performance estimates, the available MUPT data were organized into 10 cross-validation pools, constructed to preserve the underlying groupings of the data collection process. These pools were constructed by iterating down the lists of NSAP and normative studies, placing data from each subsequent study into the next cross-validation pool in round-robin fashion. This strategy ensures that all of the MUPTs collected from the same muscle remained together for purposes of aggregation as described below, while also ensuring that each pool contained studies from both Normative and NSAP characterized data. Enforcing the presence of data from both characterization classes in all testing sets controlled potential bias arising from the fact that there are significantly more normative than NSAP contractions in the training data.

The cross-validation pools where then used to construct experimental data sets such that the data in each pool were used only once for testing, with training data obtained by combining all other pools. Results were calculated across all pools, allowing average performance to be calculated. In light of the discussion in [38] and [39], full leave-one-out cross-validation was not used, as the cited works indicate that 10-fold cross-validation should provide an estimate of performance with less bias that that provided by full leave-one-out cross-validation.

### Classifier construction

A discriminant function providing the minimum-error-rate for two classes may be represented as(1)

This encodes a distance measure (δ) that provides the minimum error rate discriminant for class *k *of some K total classes for a given input vector, **x**, given the conditional probability of the observation of **x **given class *ω*_*k *_as well as the overall *a priori *probability of occurrence of samples from class *ω*_*k*_. Here we make no assumption regarding class probabilities, and assume that all *ω*_*k *_are equally probable.

If the distribution of feature values follows a Gaussian distribution, then a Bayesian discriminant function provides optimal separation between classes [[40] pp. 37-41], and a "Normal Density Discriminant Function" (NDDF) classifier may be constructed using(2)

where

in which the variables **S**_*k*_, **m**_*k *_and P(ω_*k*_) refer, respectively, to our estimates of the covariance matrix and mean vector and relative probability of occurrence of class *k *of K classes (in this case, K = 2: Normative and NSAP). In the above equations, X^-1 ^indicates the matrix inverse operation, and |*X*| indicates the calculation of the determinant.

This formulation is simply the discriminant function constructed from (1) using the general multivariate normal density(3)

in which *d *is the dimensionality (the number of input features) in the problem. As can be seen in (2), this factor drops out in the construction of the discriminant through the application of the natural logarithm.

The discriminant of (2) can therefore be seen as providing a measure of similarity to a Gaussian distribution, and is therefore equivalent to calculating the relative distance to each mean using the Mahalanolbis distance(4)

In (4), *r *provides the distance from the mean of a Gaussian (Normal) distribution in units of standard deviation, implying that the Mahalanolbis distance may then be directly used as a z-score to relate a given point to its expected probability of occurrence in the related distribution. In fact this produces the same classification results as (2).

In order to apply the above equations, the mean and covariance are calculated using all of the MUPTs available for training separated by class. The per-class mean and covariance may then be calculated directly from these groups. Mean values were calculated individually for each feature; covariance data was calculated using these per-feature means.

As mentioned above, the relative probability of occurrence of each class, **P**(*ω*_*k*_), was set to 0.5 (or "no information") to establish a uniform prior probability estimate.

### Aggregation of classifier results

Applying the NDDF classifier as described will produce an estimate of the characterization for each MUPT. Such a characterization does not take into account the fact that further information is available, specifically that MUPTs collected from the same muscle may be considered as a set in order to produce a muscle characterization, in which each MUPT supports (or refutes) a specific characterization of that muscle. Individual MUPTs can be considered to be associated with information that is meaningful only in the collective sense; by collecting such information together; it is possible to use aggregation to account for the presence of normative MUPTs in NSAP data.

Further, the characterization of individual MUPTs is not as meaningful as the characterization of a muscle as a whole. This implies that while individual MUPTs collected from a single contraction may, or may not, show indications of NSAP that may be preferentially affecting only some motor units of a muscle, it is the overall diagnosis of NSAP that need concern us here. If there is indeed such variable expression of disease state, aggregation of the individual MUPT outcomes should allow an overall diagnosis to be made, in spite of this variation in outcome associated with the individual MUPT samples.

We must be careful to form an aggregation that correctly reflects the information presented by each MUPT, without overstating the importance of any single measurement. Essentially we expect to see both MUPTs that "look normative" in muscle studies from patients with NSAP, and we expect to see MUPTs that appear consistent with NSAP in muscle studies from control subjects.

We wished to integrate the information present in a set of MUPTs sampled from the same muscle over a set of contractions into a single muscle characterization. Specifically, we wished to consider the set of MUPT results as a group of input values for some form of aggregation classifier. We therefore compared results in terms of successful muscle level characterization using four different aggregation schemes as described below.

### Independent MUP analysis

The first calculation done examines the results of the NDDF classifier as run independently on each MUPT, producing a total of 1434 characterizations. This analysis was performed for two reasons: the accuracy of the classification system when no muscle-level knowledge is used provides the minimum accuracy we would expect from aggregation, and additionally, it is these NDDF measures that will be used to produce the aggregate results to be compared.

### Vote-based aggregation

A simple and obvious aggregation strategy to aggregate the 1434 MUPT results into descriptions of the 57 muscular studies is to apply a simple majority vote scheme. We therefore simply examine all MUPTs sampled from a muscle and count, for each class, the number of MUPTs for which that class was indicated as having a maximum conditional probability. The class label that had the majority count was then applied to all MUPTs in the contraction. In cases of a tie, one of the labels was randomly chosen.

Note that this strategy does not take into account the magnitude of the difference in conditional probability used to choose the winning class; the smallest of margins produces a vote of the same weight as a unity probability.

### Bayesian aggregation

The magnitude of difference in probability may be further taken into account through further leveraging of our assumption that the class distributions may be defined as conditional probability distributions following a Gaussian curve, and using the relative probabilities found in an aggregate calculation of the joint probability of association across all MUPTs studied.

This may be easily calculated once we realize that the formulation of (1) allows us to combine the joint probabilities of observation of several **x **values, as it is equivalent, within a scale factor, of either(5)

In particular, the second formulation here indicates that in order to produce an aggregation of the joint probabilities across a series of MUPT samples **x**_1_, **x**_2_,... **x**_*n*_, we may simply multiply together all of the *δ*_*k *_values obtained for each sample within the same class to obtain an estimate of the joint probability Δ_*k*_, *i.e.;*(6)

As the normalization required to turn (6) into a true probability is the same for each class considered, it need not be considered when constructing the aggregate discriminant, as its effect will simply be to scale each probability by the same value. To calculate a *relative *probability therefore we need simply multiply the values for each discriminant obtained from (2) as shown in (6) without a need to normalize the result. We will then use the highest Δ_*k *_value to indicate the class association.

### Mean NDDF discriminant

As a final strategy, a mean distance across all MUPTs in a contraction was calculated for a given class, by calculating an average of the distances determined by the NDDF classifier. This mean value was then computed for each class, resulting in a measure describing the average distance of the MUPTs in a given contraction from each class. The contraction was then assigned to the "closest" class based on this average distance.

## Results

### Sample demographic information

The demographic information of both samples is presented in Table 2. The clinical questionnaire and clinical evaluation outcomes for the NSAP group are presented in Table 3. The upper limb tension test with radial bias (ULTT3) revealed that none of the NSAP subjects had a positive test.

**Table 2:** Demographic Data.

	n	NSAP Mean ± SD	n	Control Mean ± SD
Height (cm)	17	164.6 ± 7.9	40	170.2 ± 8.4
Weight (lbs)	17	159.2 ± 29.7	40	149.1 ± 24.2
Age (years)	17	50 ± 9**	40	27 ± 5*
MVC (N)	17	127.1 ± 48.8**	40	195.1 ± 51.3*

**Table 3:** Clinical evaluation outcomes from the Disability of arm shoulder and hand (DASH) questionnaire, SF-36 eight domain scores, ULTT3 (number of positive tests, pain threshold scores (values in brackets are normalized to third nail bed; D3), grip and pinch-grip strength for the NSAP group.

	n	NSAP Mean ± SD
DASH		
Disability score	16	23.83 ± 12.96
Work module	15	35.22 ± 31.59
Sport/art module	9	68.06 ± 29.22

SF-36		
Physical functioning	15	82.00 ± 18.01
Role physical	16	62.50 ± 38.76
Bodily pain	16	57.38 ± 18.75
General health	16	73.12 ± 20.04
Vitality	16	61.56 ± 18.86
Social functioning	16	84.38 ± 17.38
Emotional role	16	83.33 ± 32.20
Mental health	16	76.50 ± 16.58

ULLT3 (n positive)	16	0

Pain Threshold (kg/cm^2^)		
D3	16	12.87 ± 5.95
ECRB	16	5.78 ± 3.49 (45%)
FCR	16	9.18 ± 5.06 (71%)
BB	16	9.08 ± 4.74 (71%)
TB	16	8.28 ± 5.02 (64%)

Grip strength (kg)	16	33.95 ± 13.06
Pinch grip strength (kg)	16	9.41 ± 3.89

### Distribution parameter estimate stability

Table 4 reports the variability of the mean and coefficient of variation for each of the features described in Table 1.

**Table 4:** Distributions Obtained of Features Studied.

		Normative				NSAP				
	Feature	μ(μ)	σ(μ)	μ(σ)	ψ	μ(μ)	σ(μ)	μ(σ)	ψ	*t*
log	Ampl	5.923	0.555	0.016	35.361	5.883	0.485	0.018	27.127	0.17
	Duration	9.742	4.861	0.071	68.147	9.190	2.877	0.142	20.316	0.31
	Phases	2.570	0.923	0.024	39.074	2.767	0.925	0.035	26.561	0.48
	Turns	3.381	1.658	0.043	38.566	3.101	1.462	0.059	24.915	0.40

log	AAR	0.237	0.393	0.007	56.585	0.333	0.362	0.017	21.244	0.57
log	Mac Ampl	4.187	0.766	0.032	24.010	4.018	0.605	0.048	12.721	0.55
log	Mac -Pk Area	5.882	0.936	0.037	25.323	5.439	0.724	0.052	13.941	1.19
log	Mac -Pk Ampl	3.656	0.738	0.032	22.919	3.432	0.651	0.051	12.759	0.72

	Mac -Pk Dur	25.516	13.701	0.222	61.805	17.745	5.753	0.220	26.097	1.65
	IDI mean	69.881	14.657	0.466	31.425	72.858	15.857	0.557	28.444	0.44
	IDI std dev	9.500	4.293	0.067	63.896	8.265	5.259	0.173	30.441	0.58
	IDI cov	0.138	0.056	0.001	41.829	0.112	0.053	0.002	24.638	1.03

	IDRate	58.552	22.901	0.458	50.038	54.498	18.650	0.488	38.255	0.43
	FR	14.873	2.880	0.103	28.080	14.292	2.802	0.113	24.780	0.49
	FRMCD	0.192	0.111	0.003	44.018	0.147	0.095	0.004	24.676	0.98

The notation "log" indicates those columns whose data is log-transformed before analysis as shown in Table 1.

Columns indicated as σ(μ) contain the standard deviation of the mean values obtained over each feature in a given class, calculated over the 10 cross-validation tests. Conversely, columns marked μ(σ) show the average of the per-feature standard deviations, again independently for each feature. Together, these values may be used to get an estimate of the variability in the mean values obtained for the various Normative and NSAP distributions tested, and relate these to the variability of the distributions themselves, noting that all that is shown is the feature-independent variability, and not the inter-feature dependence found in a full covariance matrix.

To that end, the columns marked ψ show the coefficient of variation, which is the ratio of the standard deviation of the mean of a feature versus the mean variability of the feature overall, or(7)

This statistic measures the dispersion of the probability distribution of the feature values.

The final column in Table 4 is a *t *value calculated by taking the difference between the mean values and normalizing by the mean standard deviation values weighted by the degrees of freedom (d.f.) introduced by the tests, or(8)

where the number of degrees of freedom is 10, based on the 10× cross-fold validation. This measure provides a means of identifying the contribution to classification relative to the Normal classifier, but does not measure the information content of the feature if the assumption of Normal distribution is violated. Note that it is clear that no single feature, in and of itself, is sufficient to determine between Normative and NSAP values.

### Classification accuracy

Tables 5 through 8 are set up as confusion matrices describing the results of independent MUPT classification, vote based aggregation, Bayesian aggregation and mean NDDF discriminant respectively.

**Table 5:** NDDF (Independent)/10 fold cross-validation (MUPTs)

	Assigned Label			
True Label	Normative	NSAP	Totals	Accuracy/Performance
**Normative**	900	268	1168	0.771
**NSAP**	73	193	266	0.726

**Totals**	**973**	**461**		**0.559**

**Table 6:** NDDF + vote/10 fold cross-validation (contractions)

	Assigned Label			
True Label	Normative	NSAP	Totals	Accuracy/Performance
**Normative**	36	3	39	0.923
**NSAP**	2	14	16	0.875

**Totals**	**38**	**17**		**0.807**

**Table 7:** NDDF + Bayes/10 fold cross-validation (contractions)

	Assigned Label			
True Label	Normative	NSAP	Totals	Accuracy/Performance
**Normative**	34	5	39	0.872
**NSAP**	1	15	16	0.938

**Totals**	**35**	**20**		**0.817**

**Table 8:** Mean NDDF/10 fold cross-validation (contractions)

	Assigned Label			
True Label	Normative	NSAP	Totals	Accuracy/Performance
**Normative**	38	1	39	0.974
**NSAP**	5	11	16	0.688

**Totals**	**43**	**12**		**0.670**

Each table contains a header and summary row. The central rows of the table are set up in the following way:

• each row is labelled with the true characterization,

• the first two columns indicate the number of characterization with the true label into each of the possible characterization labels,

• the "Totals" column shows the number of elements in each true class, and

• "per-class accuracy" is the fraction of the elements that was correctly labelled for each class. Considering NSAP as a "positive test outcome," and Normative as a "negative test outcome", the per-class accuracies for the NSAP and Normative classes are, respectively, the estimates of the sensitivity and specificity of the classifier; the overall accuracy of the classifier is simply the sum of the per-class accuracy values divided by the number of classifications made.

The bottom of the table displays overall statistics. Totals are tallied for each column, which indicate the number of samples assigned to each target class; in the case of Table 5 these are MUPTs, in the remaining tables these are muscles.

The value at the foot of the "Per-class accuracy" column is simply the product of all of the per-class accuracy values assigned, and is termed "Performance." This was chosen as an overall performance statistic as it equally weights the contribution to overall performance by each class while providing a metric that can be used to compare the different classification schemes. It should be pointed out that although this metric is [0···1] bounded, the multiplicative relationship between the elements does mean it is non-linear (though monotonically increasing).

Table 5 indicates the results of analysis using the NDDF classifier when classifying each MUPT independently (i.e.; discarding the knowledge that for a set of MUPTs sampled from a muscle all the MUPTs come from the same muscle, and thus must have the same characterization). These results show that, as a baseline, approximately 3/4 of the individual MUPT characterizations have a maximum conditional probability that matches the true muscle characterization.

An analysis of the same underlying data is shown in Table 6, but with an aggregate label calculated using the voting aggregation as presented above. Immediately apparent from this table is the fact that aggregate decision making results in a much higher degree of accuracy: the poorest per-class accuracy is 0.875 based on best-vote-takes-all.

Bayesian aggregation provides somewhat different values, shown in Table 7, which indicates that the increase in accuracy is similar to that in the voting scheme.

Table 8, displaying the mean NDDF classification, shows that this technique is severely biased toward Normative, achieving an accuracy of roughly only 2 in 3 on NSAP data.

An analysis of the significance of these results was calculated using McNemar's test [41,42]. This test was constructed by examining the pair-wise differences between the same contractions as evaluated by each test. Four groups were constructed, containing the counts of: the instances for which both classifiers were correct; the instances for which both were incorrect; those for which there was an improvement in classification by the second classifier (*i.e.; *the first classifier was wrong, but the second was correct); and those for which there was a degradation (first was correct, the second was wrong).

The McNemar test relates the association between the changes in "treatment" (here the change in classifier) and the change in outcome (termed "discordant pairs"). With no association, the two discordant pairs should be equal, and a χ^2 ^value can then be calculated from two discordant pairs α and β using(9)

calculated using 1 degree of freedom.

Table 9 provides the number of improved and degraded discordant pairs, as well as the 2-tailed *p*-value and, χ^2 ^. As can be seen in this table, there are no significant differences between any groups in these data.

**Table 9:** McNemar Test Results on Classifier Performance

Classifiers	Improved	Degraded	2-tailed *p*-value	χ^2^	Odds Ratio	Confidence Interval
MICD+Bayes -vs- MICD+mean	4	4	0.72	0.125	1.00	0.189 ... 5.37
MICD+Bayes -vs- MICD+vote	2	1	1.00	0.125	2.00	0.104 ... 118
MICD+vote -vs- MICD+mean	4	3	1.00	0.000	1.333	0.226 ... 9.10

## Discussion

The clinical assessment showed there were no strength differences between the individuals with and without NSAP; in fact the groups were very similar other than the fact that the individuals with forearm pain scored higher on measures of pain and disability, had a lower tolerance to pressure applied to their ECRB muscle and their triceps muscle. Other than non-specific symptoms of pain, therefore, there were no features on examination that would suggest that the individuals with NSAP had either myopathy or neuropathy.

### Classification outcome

The power of Bayesian aggregation would lead us to expect that the results in Table 7 would provide a significantly higher performance than the simple voting results shown in Table 6. The fact that this is not the case is very instructive regarding the estimation of the underlying data distribution. Such an expectation rests upon the assumption that the Bayesian aggregation has access to useful and correct information describing both the Normative class and the NSAP class; which in turn is based on the assumption that both of these are in fact Gaussian distributions.

The fact that muscle characterization based on individual MUPT characterizations performed quite well (*i.e*., 75% accuracy on MUPT analysis) lends a great deal of support to this premise, as poor results are found when using this classification scheme on significantly skewed distributions. The evidence here is that although the distributions are centrally limited, the assumption of a Gaussian distribution is not well founded in this case, though the limitations of this assumption are not severe.

One potential weakness stems from the amount of data available to estimate distribution parameters. Although the method of estimation used is optimal given a Gaussian distribution [[40], pp. 36], insufficient data will provide an unstable estimate. The stability of our parameter estimates as shown in Table 4 indicate not only that the mean values calculated are relatively stable, but that the variance in these estimates are significantly smaller than the per-feature standard deviations associated with each feature.

Essentially, the conclusion that may be reached based on our observations is that although the Bayesian aggregation technique using sets of MUPTs substantially increases classification accuracy (relative to unaggregated data), the assumption of a Gaussian distribution to describe the data limits its effectiveness; there is no more information, on average, available in the estimate of distribution shape and Bayesian aggregation than is available through aggregate voting.

The outlier detection methods introduced in [43] and applied and discussed in [44,45] are relevant here as it is exactly these outliers that contribute to the ability of the Bayesian estimator to determine that these muscles are not normative. The inference applied here limits the extent to which outlier following will be performed, ensuring that the outlier-based classifications are appropriately weighted by the observation of normative MUPTs. If appropriate probabilities are available for the Bayesian estimator, it may be expected that this will provide an excellent mechanism for determining when enough MUPTs have been observed, allowing the central question of [45] to be explored in a probabilistic sense.

This observation in turn supports the idea that with a better understanding of the true data distribution, a better Bayesian estimator may be produced. The authors intend to apply an event-based treatment introduced in earlier work [36] to these data, providing an analysis that is free from the assumption of a Gaussian distribution.

The measure of stability (column marked ψ in Table 4) provides insight into the variability of the means of the two classes relative to the class variances; when compared with the *t *values shown in the right-most column of Table 4 we see that there is significant information present in these columns. We may therefore conclude that though our assumption of a Gaussian distribution does not accurately reflect the underlying distribution of the data for all of the reasons mentioned above, there is significant information content in these data that will allow decisions to be made. Further, we can estimate, based on these data, which features are likely to be the most informative overall, and further exploration of these features in a non-parametric analysis is warranted.

We intend therefore to proceed to an analysis of these data using stronger, non-parametric techniques to ascertain whether a better classification may be obtained then the already-strong performance obtained here.

## Conclusions

In support of the findings of [28,29], MUPT classification performance may be improved through the application of Bayesian aggregation, however the degree of improvement may be limited to that obtained through simpler means, such as majority vote.

The limitation in the performance improvement observed is not related to an inability to estimate distribution parameters because of a lack of data; instead, limitations are apparent because of assumptions of distribution shape that do not reflect the true MUP morphological and MU firing pattern changes occurring due to involvement.

An investigation into more appropriate methods to examine the distribution of involved versus normative data should allow Bayesian aggregation to achieve improved characterization accuracy.

## Competing interests

The authors declare that they have no competing interests.

## Authors' contributions

All authors consulted and collaborated throughout the study. LMcL conceived of the initial idea, while AH-W carried out the experimental procedure, developed all related Matlab^© ^programs and drafted the manuscript. KMC collected the data and performed initial statistical analyses. All authors participated in the study design, and read and approved the final manuscript.
